# Supplementary material for: Morphological Characteristics of Genital Organ-Associated Lymphoid Tissue in the Vaginal Vestibule of Goats and Pigs
Source: Vet Sci. 2023 Jan 11;10(1):51. doi: 10.3390/vetsci10010051 (PMC9864709; doi:10.3390/vetsci10010051)
Supplement: Supplementary file 1 [file vetsci-10-00051-s001.zip › Supplementary Figure S1.pdf]

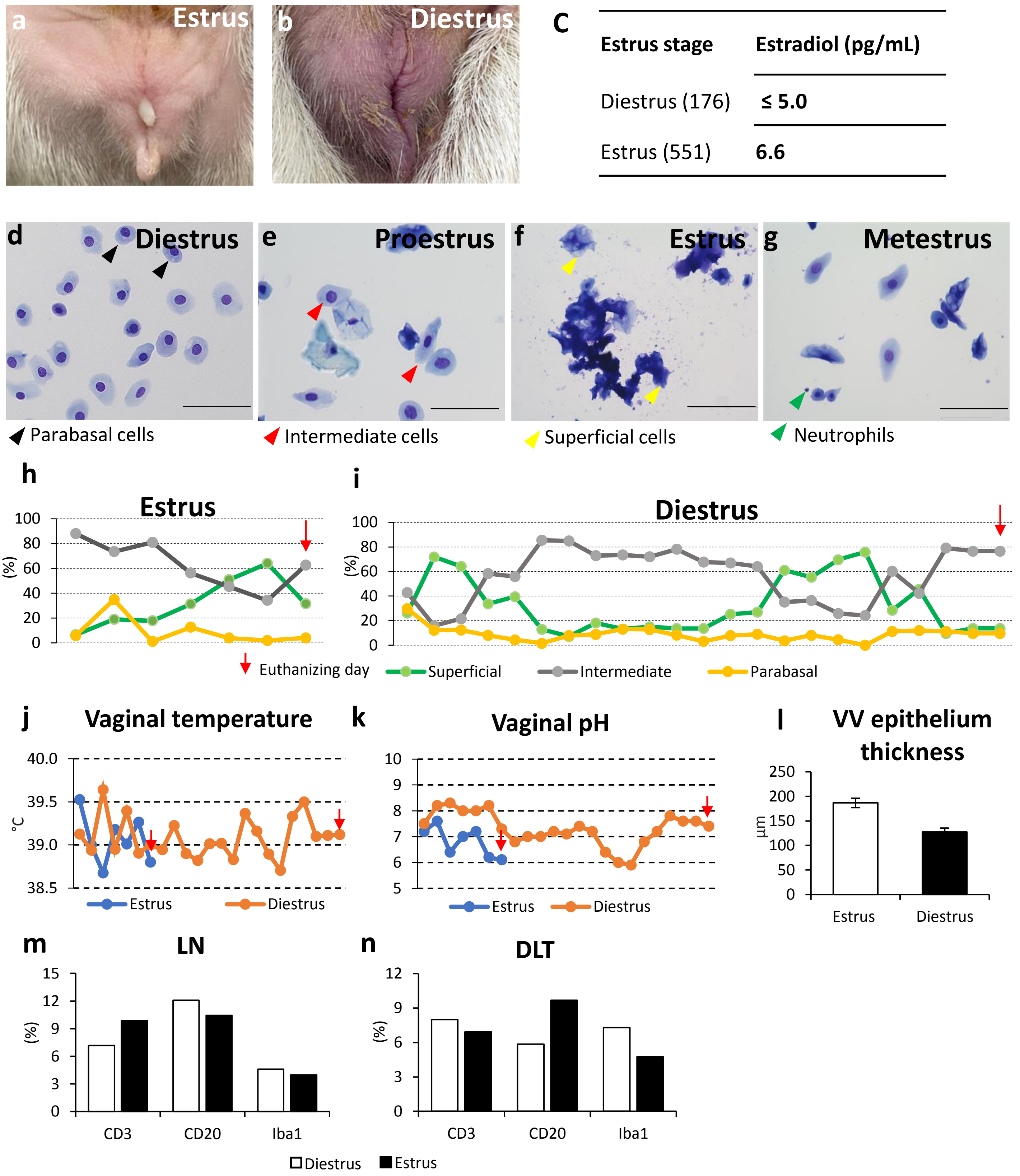

Supplemental Figure S1. Immune cell composition in different stages of the estrus cycle

(**A**) Vulvar condition during the estrus stage, showing the discharge of sticky cloudy mucous, swelling, and redness. (**B**) Vulva condition during the diestrus stage. (**C**) Estradiol level in the blood serum of goats in different stages of the estrus cycle. (**D, E, F, G**) Vaginal cytology in diestrus, proestrus, estrus, and metestrus stages. Scale bars = 50 μm. (**H, I**) Daily percentage of exfoliative vaginal cells of goats expected as estrus (H) and diestrus (I) stages. (**J**) Daily vaginal temperature of goats. (**K**) Daily vaginal pH of goats. (**L**) Epithelium thickness of VV in goats expected as estrus and diestrus stages. Values = mean ± SE. *n* = 1. (**M**) Percentage of immune cells occupying the LF during the estrus and diestrus stages. *n* = 1 in each stage. (**N**) Percentage of immune cells occupying the DLT during the estrus and diestrus stages. *n* = 1 in each stage. VV: vaginal vestibule, DLT: diffuse lymphoid tissue, LF: lymphatic follicle.
