# Supplementary material for: Morphological Characteristics of Genital Organ-Associated Lymphoid Tissue in the Vaginal Vestibule of Goats and Pigs
Source: Vet Sci. 2023 Jan 11;10(1):51. doi: 10.3390/vetsci10010051 (PMC9864709; doi:10.3390/vetsci10010051)
Supplement: Supplementary file 1 [file vetsci-10-00051-s001.zip › Supplementary Figure S2.pdf]

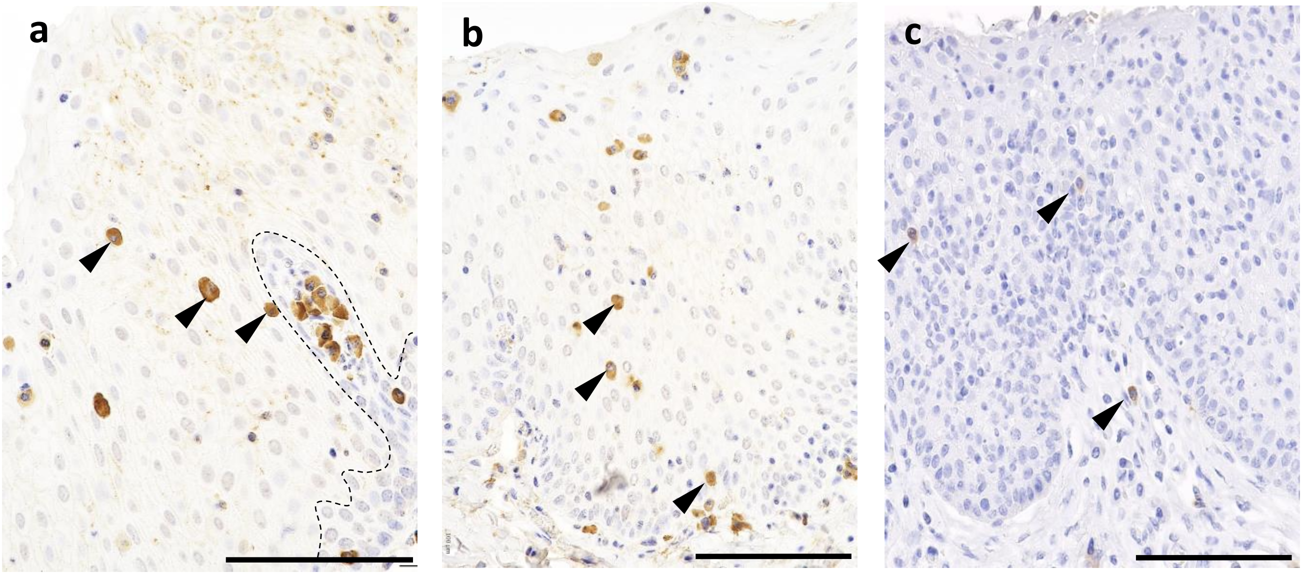

Supplemental Figure S2. IgA$^+$ plasma cell localization in the epithelium of VV. (**A, B**) Goat IgA plasma cells in the epithelium of VV. (**C**) Pig IgA plasma cells in the epithelium of VV proportion of exfoliative vaginal cells during the stages of the estrus cycle using vaginal smear techniques. Scale bars = 100 μm.
