# Supplementary material for: Morphological Characteristics of Genital Organ-Associated Lymphoid Tissue in the Vaginal Vestibule of Goats and Pigs
Source: Vet Sci. 2023 Jan 11;10(1):51. doi: 10.3390/vetsci10010051 (PMC9864709; doi:10.3390/vetsci10010051)
Supplement: Supplementary file 1 [file vetsci-10-00051-s001.zip › Supplementary table S1.pdf]

Supplemental Table S1. List of antibody and conditions used for immunohistochemistry

Parameters	Antigen Retrieval	Blocking	Primary Antibody	Secondary Antibody
CD20	TB 115°C, 15 min	10% NGS	Rabbit polyclonal antibodies (Spring Bioscience) 1:300	Goat anti-rabbit (SABPO kit, Nichirei)
CD3	CB 115°C, 15 min	10% NGS	Rabbit polyclonal antibodies (Nichirei) 1:200	Goat anti-rabbit (SABPO kit, Nichirei)
Iba1	CB 115°C, 15 min	10% NGS	Rabbit polyclonal antibodies (Wako) 1: 1200	Goat anti-rabbit (SABPO kit, Nichirei)
PNAd	CB 115°C, 15 min	10% NGS	Rat polyclonal antibodies (Biolegend) 1: 200	Goat anti-rat IgG (Biolegend) 1:100 (Biotinylated)
IgA	TB 115°C, 15 min	10% NGS	Rabbit polyclonal antibodies (Bethyl) 1: 100	Goat anti-rabbit (SABPO kit, Nichirei)
IgG	CB 115°C, 15 min	10% NGS	Rabbit polyclonal antibodies (Bethyl) 1: 100	Goat anti-rabbit (SABPO kit, Nichirei)
Langerin	TB 115°C, 15 min	10% NGS	Rabbit polyclonal antibodies (Proteintech) 1: 50	Goat anti-rabbit (SABPO kit, Nichirei)

CB: citrate buffer pH6, TB: tris buffer pH9, and NGS: normal goat serum
