# Supplementary material for: Morphological Characteristics of Genital Organ-Associated Lymphoid Tissue in the Vaginal Vestibule of Goats and Pigs
Source: Vet Sci. 2023 Jan 11;10(1):51. doi: 10.3390/vetsci10010051 (PMC9864709; doi:10.3390/vetsci10010051)
Supplement: Supplementary file 1 [file vetsci-10-00051-s001.zip › Supplementary table S2.pdf]

Supplemental Table S2. List of antibody and conditions used for immunofluorescence

Parameters	Antigen Retrieval	Blocking	Primary Antibody	Secondary Antibody
IgA	TB 115°C, 15 min	5% NDS	Rabbit polyclonal antibodies (Bethyl) 1: 100	Donkey anti-rabbit (SABPO kit, Nichirei)
IgG	CB 115°C, 15 min	5% NDS	Rabbit polyclonal antibodies (Bethyl) 1: 100	Donkey anti-rabbit (SABPO kit, Nichirei)

CB: citrate buffer pH6, TB: tris buffer pH9, and NDS: normal donkey serum
